# A Shared Cancer Follow-Up Model of Care Between General Practitioners and Radiation Oncologists for Patients With Breast, Prostate, and Colorectal Cancer: Protocol for a Mixed Methods Implementation Study

**DOI:** 10.2196/21752

**Published:** 2021-01-19

**Authors:** Tiffany Sandell, Heike Schütze, Andrew Miller

**Affiliations:** 1 Wollongong Hospital Wollongong Australia; 2 University of Wollongong Wollongong Australia; 3 University of New South Wales Sydney Australia

**Keywords:** radiation oncology, general practice, health technology, communication, cancer, shared care, follow-up

## Abstract

**Background:**

The rising incidence of cancer and increasing numbers of cancer survivors have resulted in the need to find alternative models of care for cancer follow-up care. The acceptability for follow-up care in general practice is growing, and acceptance increases with shared-care models where oncologists continue to oversee the care. However, a major barrier to this model is the effective exchange of information in real time between oncologists and general practitioners. Improved communication technology plays an important role in the acceptability and feasibility of shared cancer follow-up care.

**Objective:**

The aim of this study is to evaluate the feasibility and acceptability of a shared cancer follow-up model of care between patients, general practitioners and radiation oncologists.

**Methods:**

This is a mixed methods, multisite implementation study exploring shared follow-up care for breast, colorectal, and prostate cancer patients treated with curative radiotherapy in New South Wales, Australia. This study uses web-based technology to support general practitioners in performing some aspects of routine radiotherapy follow-up care, while being overseen by a radiation oncologist in real time. The study has two phases: Phase 1 is designed to establish the level of agreement between general practitioners and radiation oncologists and Phase 2 is designed to implement shared follow-up care into practice and to evaluate this implementation.

**Results:**

Recruitment of radiation oncologists, patients, and general practitioners commenced in December 2020 and will continue until February 2021. Data collection will occur during 2021, and data will be ready for analysis by the end of 2021.

**Conclusions:**

Few studies have investigated the role of health technologies in supporting communication deficiencies for shared cancer follow-up care. The implementation and evaluation of models of care need to be conducted using a person-centered approach that is responsive to patients’ preferences and needs. Should the findings of the study be acceptable and feasible to radiation oncologists, general practitioners, and patients, it can be quickly implemented and expanded to other tumor groups or to medical oncology and hematology.

**Trial Registration:**

Australian New Zealand Clinical Trials Registry ACTRN12620001083987; http://www.anzctr.org.au/Trial/Registration/TrialReview.aspx?id=380057

**International Registered Report Identifier (IRRID):**

PRR1-10.2196/21752

## Introduction

The increasing incidence of cancer, coupled with improved survivorship, has resulted in higher demand for cancer follow-up care [1-3]. This has led to the sustainability of oncologist-led cancer follow-up care in the secondary health setting being questioned [4,5] and to a call for alternative models of cancer follow-up care [6,7]. There is a growing body of literature on the benefits of shared cancer follow-up models between general practitioners and oncologists [8]; however, this is yet to be integrated into routine practice.

Randomized controlled trials have shown that cancer follow-up care delivered by a general practitioner in the primary health care setting produces no difference in the rate of recurrence or quality of life compared to cancer follow-up with an oncologist [9-11]. General practitioners are willing to take a greater role in cancer follow-up care [12] provided they are supported by the oncologist [13-16] and the oncologist maintains overall responsibility [17].

Despite an acceptance by patients for their general practitioner to be involved in their follow-up care, barriers to shared care exist. The barriers are role clarification [18-20] and effective two-way communication [21-25]. There is a need for a robust information-sharing system that allows both the general practitioner and the overseeing oncologist to be involved in the follow-up care. Real-time and open access to patient information is crucial to coordinate the care of cancer survivors appropriately [26-28].

At present, cancer patients maintain follow-up with their oncologists in the secondary health care setting, and routine communication is transferred from the oncologist to the general practitioner via letter or secure email. In the case where a general practitioner has undertaken a cancer-specific follow-up, it is uncommon for the general practitioner to communicate their findings to the oncologist. This study will trial a web-based technology to breach the communication divide between the general practitioner and the oncologist so that they can work together collaboratively, should patients choose a shared-care model.

To our knowledge, there is currently no system that supports the involvement of general practitioners in shared cancer follow-up care where the radiation oncologist can oversee the care. This study trials a web-based system that allows general practitioners to undertake routine aspects of cancer follow-up care, while sharing the data with oncologists at the hospital in real time so that they can continue to monitor, oversee, and maintain responsibility for the patient.

This research aims to evaluate the feasibility and acceptability of a shared cancer follow-up model of care between patients, general practitioners and radiation oncologists. The objectives of this study are to implement a model of care using a web-based system that transfers clinical information between the general practitioner and radiation oncologist in real time, to determine the level of agreement between general practitioners and oncologists completing a standardized follow-up assessment, and to establish the feasibility and acceptability of this model of care.

## Methods

### Study Design

This research is a mixed methods, multisite implementation study for breast, colorectal, and prostate cancer patients who have undertaken curative radiotherapy treatment. Mixed methods investigations involve integrating quantitative and qualitative data collection and analysis into a single study [29] and can strengthen the credibility of evidence and evaluation [30].

**Figure 1:**
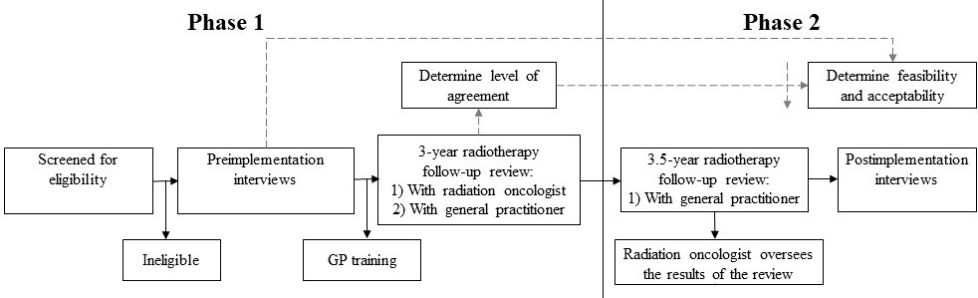
Flow diagram of study phases. GP: general practitioner.

The study will implement the shared cancer follow-up model of care into practice at baseline (Phase 1) and at 6 months postrecruitment (Phase 2) (see Figure 1). During Phase 1, there will be a standard clinical review by the radiation oncologist as per the patient's routine follow-up schedule, plus an additional follow-up review by the general practitioner using the same standardized follow-up assessment. This first phase will determine the level of agreement between general practitioners and radiation oncologists when completing the same radiotherapy follow-up clinical assessment on the patient. This first phase is essential, as it informs the educational and training requirements for general practitioners. By demonstrating the level of agreement, it reassures both the general practitioner and radiation oncologist that the general practitioner can reliably conduct a cancer-specific follow-up review.

The second phase of the study is the implementation of the shared cancer follow-up model of care into practice. The patient will visit their general practitioner at 3.5 years follow-up for a radiation oncology–specific follow-up appointment. The results will be transferred to the hospital, and the patient's radiation oncologist will be alerted by an automatic quality checklist to review the outcomes of the review in real time on the hospital's oncology information system. The system has a rapid referral built into it in the case of adverse events or should the general practitioner suspect cancer recurrence.

### Study Setting

The research will be conducted within the Illawarra Shoalhaven Local Health District (ISLHD) region in New South Wales, Australia. The ISLHD provides public health services to over 400,000 people and cancer services to almost 9000 people annually (ie, medical oncology, hematology, and radiation oncology). Radiation oncology outpatient services are provided at Wollongong Hospital (ie, tertiary hospital and regional care) and the Shoalhaven District Memorial Hospital (ie, secondary hospital and rural care).

The ISLHD radiation oncology service consults and treats over 1400 patients with radiotherapy and conducts over 5000 follow-up consultations annually. The service has experienced a 20% increase in follow-up consultations over a 5-year period (2015-2019), and treatment activity is projected to increase by 18% by 2031. A substantial proportion of radiotherapy treatment at each site is attributed to breast, colorectal, and prostate cancer.

The study will take place at the two hospital radiation oncology outpatient clinics and in the referring general practices. The relationship between general practice and local health districts in Australia is increasingly pivotal to the health system. General practice in Australia is typically comprised of small businesses with an average of three to five general practitioners, and a universal medical insurance scheme (ie, Medicare) covers all or part of a person’s cost to visit a general practitioner [31].

### Local Follow-Up Guidelines

While there are many statements regarding “standard follow-up practices,” postradiotherapy follow-up for patients varies greatly depending on the disease type, the oncologist's preference, and the patient's preference. At the ISLHD, a visit 6 weeks after radiotherapy is routine for most cases to review the settling of acute side effects. The pattern of remaining follow-up sessions for all cancers will include a period of every 3 months for the first year and every 6 months for the second year, followed by yearly reviews and then, finally, discharge from follow-up. For many cancers, a 5-year period of follow-up is common.

At the ISLHD, an acceptable practice for breast cancer patients’ postradiotherapy follow-up care would be a follow-up at 6 weeks, then every 3 months for 2 years, then every 6 months to 5 years, and then yearly to 10 years. An acceptable practice for colorectal cancer patients would be follow-ups every 6 months for the first year and then yearly to 5 years. An acceptable practice for prostate cancer patients would be follow-ups every 6 months or yearly to 5 years. However, the actual frequency depends on the individual patient's health, stage, and treatment and their preference for whom to see; in addition, there is currently no early discharge, transfer of care, or shared care for radiation oncology follow-up care to general practitioners.

### Health Technology

The free and open source software framework PROsaiq (Didymo Pty Ltd) will be used [32]. PROsaiq is based around a web server that extracts assessments from inside the oncology information system and encodes the assessment data into XForms (ie, an XML format used for collecting inputs from web forms), which is then presented as a webpage in a web browser. When the clinical assessment is completed on a smart device (ie, phone, computer, or tablet), the clinical assessment is returned to the web server and converted into a Health Level Seven (HL7) message; HL7 is an accepted international communication standard for clinical systems, such as those comprising laboratory information. The HL7 message is presented to the oncology information system MOSAIQ (Elekta AB), where it is imported to become part of the patient's oncological record.

Australia is equipped with reliable internet capability, and the webpage link will be made available to the general practitioner by integrating it into a current local system that they utilize. The general practitioner will complete the patient follow-up clinical assessments using PROsaiq, and the radiation oncologist will receive an automated alert in real time to review the results at the hospital. PROsaiq has been trialed for the collection of cancer patient–reported quality-of-life outcomes from patients and has demonstrated its operational feasibility [33].

### Eligibility Criteria

To be eligible for the study, patients must (1) have a previous diagnosis of colorectal, breast, or prostate cancer; (2) have completed curative-intent radiotherapy treatment and are due for their 3-year review; (3) be over 18 years of age; (4) be able to understand and speak English; and (5) have a general practitioner willing to participate. Patients who do not meet these criteria will be excluded, as will patients who have suspected or confirmed recurrence of cancer.

Patients 3 years posttreatment have been selected, as it was deemed a safe time period by the oncologists for a feasibility study, and the patients will have experienced the standard oncologist-led follow-up model. Participants can withdraw at any stage up until data analysis.

### Sample Size

The sample will consist of 20 triads comprising the patient, their radiation oncologist, and their general practitioner, for a total of 35 to 45 participants. A total of 10 patients will be from the Wollongong Cancer Centre (ie, regional) and 10 will be from the Shoalhaven Cancer Centre (ie, rural).

Sample size guidelines for qualitative interviews suggest that a range between 20 and 30 interviews is adequate for each group to reach data saturation [34]. The sample size for the quantitative level of agreement data requires a minimum of 5 samples; however, to increase the confidence interval, a higher sample is required [35].

### Recruitment

The radiation oncologists will review their follow-up clinic lists from both sites and screen for initial inclusion criteria. The researcher will invite each patient to participate via a postal letter on behalf of the radiation oncologist. Once each patient consents to participate, their general practitioner will be invited. General practitioners will be eligible for continuing professional development points for participating. If the general practitioners do not consent to participate, the patient will not be eligible.

### Implementation

The foundation of this shared cancer follow-up model of care is that clinician communication exchange is two-way and in real time, while the radiation oncologist continues to oversee the follow-up care. The model includes real-time transfer of results, internal system alerts, and rapid referral to address any issues that may arise. During this study, patients maintain their current specialist standard follow-up care, with all relevant specialists, and will continue follow-up care with their radiation oncologist upon completion of the study.

General practitioners will complete a standardized online radiation oncology course developed by the Cancer Institute New South Wales [36]. The course developed for health professionals addresses the principles of radiation therapy, patient assessment grading systems of side effects, and supportive care management. General practitioners will receive one-on-one training by a radiation oncologist that includes localized radiotherapy-specific follow-up care, a review of the recruited patient’s treatment background, and a demonstration of the clinical follow-up assessment that the general practitioner will use in the patient's follow-up review.

### Data Collection

#### Overview

The PROsaiq software will be used to administer clinical assessments. The assessments were compiled internally at the ISLHD for follow-up of radiotherapy patients. These clinical assessments review physical items on a scale from 0 to 4 for items specific to radiotherapy follow-up care, such as pain, fatigue, physical performance, bowel issues, urinary issues, and appetite (see Table 1). The included scales were sourced from the Radiation Therapy Oncology Group scales [37] and the Common Terminology Criteria for Adverse Events, version 3.0 [38].

**Table 1:** Radiation oncology follow-up standardized clinical assessment.

Tumor type	Clinical assessments
Breast	Fatigue, ECOG (Eastern Cooperative Oncology Group) Performance Status, appetite, weight loss, chest and breast pain, telangiectasia, lymphedema-related fibrosis, and disease state (ie, local, regional, or distant)
Colorectal	Fatigue, ECOG Performance Status, appetite, weight loss, proctitis, pelvic pain, vomiting, and diarrhea
Prostate	Fatigue, ECOG Performance Status, erectile dysfunction, dysuria, and rectal hemorrhage

#### Quantitative Data

The quantitative data will be collected from Phase 1. The radiation oncologist will enter the clinical assessment directly into the oncology information system, while the general practitioners will enter the clinical assessment on the webpage link that will be provided to the general practitioner. Both sets of data from these clinical assessments will be stored in the hospital oncology information system.

#### Qualitative Data

At pre- and postimplementation, participants (ie, patients, general practitioners, and radiation oncologists) will participate in semistructured interviews following a topic guide about radiotherapy follow-up care and their experience of shared care. Demographic data will be collected for all participants (ie, age, sex, level of education, and working years). The interviews will be audio-recorded and transcribed verbatim in preparation for thematic analysis in NVivo (QSR International).

### Data Analyses

#### Quantitative Data

The clinical assessment data will be extracted from the oncology information system; the Cohen κ value and percent agreement for each variable from Table 1 will determine the level of agreement between general practitioners and radiation oncologists. The agreement will assess the concordance between two measurements of each variable with the expectation that there will be near-perfect agreement on each item (>0.81). The results of the analysis and level of agreement will be presented to the general practitioners and radiation oncologists to guide any additional education and training.

#### Qualitative Data

Thematic analysis is a commonly used analytical approach for qualitative data in implementation studies [39]. This involves mapping the transcribed data and emergent themes onto a priori domains. The themes will be compared across the regional and rural sites (ie, Wollongong and Shoalhaven) and triangulated between radiation oncologists, patients, and general practitioners.

### Ethics Approval and Trial Registration

Ethics approval was received on May 12, 2020, from the Joint University of Wollongong and the ISLHD Human Research Ethics Committee (2020/ETH00301). The trial was registered with the Australian New Zealand Clinical Trials Registry on October 20, 2020 (ACTRN12620001083987).

## Results

Recruitment of radiation oncologists, patients, and general practitioners commenced in December 2020 and will continue until February 2021. Data collection will occur during 2021, and data will be ready for analysis by the end of 2021.

## Discussion

### Overview

The important skill set and experience that oncologists have is undisputed. However, there appear to be limited alternate models of cancer follow-up care that address the principles of equity in access, connecting health services, and where the cancer survivor can make an informed decision about their cancer follow-up care. Cancer survivors are more likely to accept shared cancer follow-up care with a general practitioner if their care is overseen by their oncologist [15]. However, effective two-way communication between oncologists and general practitioners is lacking. Improved communication is the strongest enabler to routine shared cancer follow-up care and is an area that is still being established [22,40-42].

Few studies have investigated the role of health technologies in supporting communication deficiencies for shared cancer follow-up care [43]. There have been no explicit recommendations of what type of health technology to use or how to use it. Health technology has been embraced for the collection of patient-reported outcomes of cancer patients during follow-up care, which utilizes the internet to complete online assessments that connect to the hospitals’ patient medical files [44]. To our knowledge, using this type of technology between general practitioners and the oncologists is the first of its kind.

The body of literature on the benefits of general practitioner–led and shared cancer follow-up models of care is growing. Although shared follow-up care may not be desired or appropriate for everyone, Australia’s oncologist-led model currently leaves limited patient choice as to when, where, and by whom their follow-up care is delivered. A well-informed patient can actively participate in the decision-making process about their care based on their personal circumstances, beliefs, and priorities.

Oncologists, general practitioners, and patients are supportive of a model of shared care [15,16,45]; however, any model developed needs to address the two-way communication barrier and be evaluated for acceptability [46]. The outcomes of this study may lead to a longitudinal implementation to measure patient satisfaction, cost-benefit analysis, health economic analysis, management of rapid referrals, and long-term outcomes of patients.

### Limitations

Possible limitations of this research are the number of participants needed to determine the level of agreement; the research team will monitor this. Another limitation identified is that the general practitioners and radiation oncologists recruited may assess the same oncological patients from a different viewpoint due to differences in training. The researcher will assist in the coordination of appointments and try to minimize the impact on the patients and health professionals.

## Abbreviations

HL7: Health Level Seven
ISLHD: Illawarra Shoalhaven Local Health District
